# Construction and Comprehensive Prognostic Analysis of a lncRNA–miRNA–mRNA Regulatory Network and Tumor Immune Cell Infiltration in Colorectal Cancer

**DOI:** 10.3389/fgene.2021.652601

**Published:** 2021-07-01

**Authors:** Xiong Guo, Xiaolong Liang, Yujun Wang, Anqi Cheng, Chuan Qin, Han Zhang, Ziwei Wang

**Affiliations:** ^1^Department of Gastrointestinal Surgery, The First Affiliated Hospital of Chongqing Medical University, Chongqing, China; ^2^Department of Pathology, Daping Hospital, Army Military Medical University, Chongqing, China; ^3^Department of Gastrointestinal Surgery, Three Gorges Hospital, Chongqing University, Chongqing, China; ^4^Department of Digestive Oncology, Three Gorges Hospital, Chongqing University, Chongqing, China

**Keywords:** colorectal cancer, ceRNA network, infiltrating immune cells, prognostic model, bioinformatics analysis

## Abstract

Colorectal cancer (CRC) is a malignant tumor with high morbidity and mortality worldwide. Recent studies have shown that long noncoding RNAs (lncRNAs) play an important role in almost all human tumors, including CRC. Competitive endogenous RNA (ceRNA) regulatory networks have become hot topics in cancer research. Tumor-infiltrating immune cells (TICs) have also been reported to be closely related to the survival and prognosis of CRC patients. In this study, we used the lncRNA–miRNA–mRNA regulatory network combined with tumor immune cell infiltration to predict the survival and prognosis of 598 CRC patients. First, we downloaded the lncRNA, mRNA, and miRNA transcriptome data of CRC patients from The Cancer Genome Atlas (TCGA) database and identified differentially expressed genes through “limma” package of R software. The ceRNA regulatory network was established by using the “GDCRNATools” R package. Then, univariate Cox analysis and least absolute shrinkage and selection operator analysis were performed to identify the optimal prognostic network nodes, including SRPX, UST, H19, SNHG7, hsa-miR-29b-3p, and TTYH3. Next, we analyzed the differences in 22 types of TICs between 58 normal subjects and 206 CRC patients and included memory CD4 T cells, dendritic cells and neutrophils in the construction of a prognostic model. Finally, we identified the relationship between the ceRNA prognostic model and the infiltrating immune cell prognostic model. In conclusion, we constructed two prognostic models that provide insights on the prognosis and treatment strategy of CRC.

## Introduction

Colorectal cancer (CRC) is one of the most common malignant tumors that seriously threatens human health. Approximately 1.9 million new cases occur worldwide each year, and approximately 935,000 people die of CRC, which has become the second leading cause of cancer death (Sung et al., 2021). In the last decade, progress has been made in the diagnosis and treatment of CRC, but the overall survival (OS) rate of patients has not improved significantly. Prognostic biomarkers can be used for disease stratification, which can also help formulate treatment strategies for CRC.

At present, the common biomarkers such as kirsten rat sarcoma viral oncogene homolog (KRAS), v-raf murine sarcoma viral oncogene homolog B (BRAF), microsatellite stability (MSI), mismatch repair deficiency (dMMR), and carcinoembryonic antigen (CEA) were wildly used to guide clinical practice (Koulis et al., 2020). In addition, epigenetic alterations are involved in CRC carcinogenesis (Picardo et al., 2019; Rodriguez-Casanova et al., 2021). A various of genes involved epigenetic alterations were identified as potential prognostic biomarkers or therapeutic targets in recent years (Picardo et al., 2019). In particular, some drugs targeting epigenetic modifications were developed astherapeutic molecularagainst CRC (Patnaik and Anupriya, 2019). Among these epigenetic alterations, preliminary studies have demonstrated that non-coding RNAs such as long noncoding RNAs(lncRNAs) and miRNAs play crucial roles in prognosis and diagnosis of CRC (De Robertis et al., 2018; Chai et al., 2020; Yu et al., 2021).

Long noncoding RNAs are defined as noncoding RNAs longer than 200 nucleotides, and they have been reported to be abnormally expressed in various types of cancer cells and play a vital role in the occurrence and progression of cancer (Xiao et al., 2018; Li et al., 2019; Ni et al., 2019; Wei et al., 2019). In addition, lncRNAs are considered effective diagnostic biomarkers in cancer (Chao and Zhou, 2019; Xu et al., 2020). In recent years, many studies have elaborated the function of the lncRNA-mediated competitive endogenous RNA(ceRNA) network in the progression of many cancers (Le et al., 2019; Long et al., 2019). The ceRNA network has also been reported to be related to CRC (Wang et al., 2019). However, most of these studies have not established a prognostic model for CRC.

Mounting evidence indicates that the occurrence of tumors is a complex and dynamic process associated with various cellular and noncellular factors within the tumor microenvironment (TME) (Belli et al., 2018; Terrén et al., 2019). The TME can regulate cancer progression (Lee and Cheah, 2019), and cancer-infiltrating immune cells within this environment have been reported to have value in predicting cancer prognosis (Krempski et al., 2011). Infiltrating immune cells have been used as prognostic biomarkers for various cancers (Jochems and Schlom, 2011; Ladányi, 2015; Liu et al., 2017). Studies focusing on infiltrating immune cells help to predict the prognosis of patients and formulate more effective treatment strategies.

Systematic research has not been performed for the ceRNA network and tumor immune microenvironment in large-scale cohorts with CRC. In this study, we constructed a ceRNA network prognostic model and tested the accuracy of the model. Then, we established an infiltrating immune cell prognostic model that also functions in CRC patients. In addition, we explored the relationship between the ceRNA prognostic model and the infiltrating immune cell prognostic model.

## Materials and Methods

### Data Acquisition and Processing

The transcriptome data of CRC patients were downloaded from the TCGA-COAD and TCGA-READ projects in the Cancer Genome Atlas(TCGA) database, including mRNA, lncRNA, and miRNA expression data. Matching clinical information was also downloaded from the database, including the patient’s age, sex, survival time, and survival state. Patients who survived less than 30 days and had incomplete clinical information were excluded. The clinical characteristics of patients with colorectal cancer shown in Table 1.

**TABLE 1:** Clinical characteristics of patients with colorectal cancer (CRC) in The Cancer Genome Atlas (TCGA).

Characteristic	629 CRC patients
Age	
<65	253 (40.22%)
≥65	376 (59.78%)
Gender	
Female	294 (46.74%)
Male	335 (53.26%)
Stage	
Stage I	109 (17.32%)
Stage II	229 (36.41%)
Stage III	181(28.76%)
Stage IV	90 (14.31%)
Unknown	20 (3.18%)
TNM-T	
T1	20 (3.185)
T2	109 (17.32%)
T3	428 (68.04%)
T4	70 (11.13%)
Unknown	2 (0.32%)
TNM-N	
N0	357(56.76%)
N1	151(24.01%)
N2	118(18.76%)
NX	2 (0.32%)
Unknown	1 (0.16%)
TNM-M	
M0	467 (74.24)
M1	89 (14.25%)
MX	64 (10.17%)
Unknown	9 (0.14%)

*NX and MX, not available.*

Immune infiltration data were collected from the CIBERSORT^1^ database, which contains abundance data for 22 types of tumor-infiltrating immune cells: plasma cells, memory B cells, naive B cells, CD8 T cells, resting memory CD4 T cells, activated memory CD4 T cells, naive CD4 T cells, regulatory T cells, follicular helper T cells, resting NK cells, gamma delta T cells, monocytes, activated NK cells, M2 macrophages, M1 macrophages, M0 macrophages, resting dendritic cells, resting mast cells, eosinophils, activated dendritic cells, neutrophils, and activated mast cells. We combined the TCGA-ID of CRC patients with the patient’s immune cell abundance to obtain the immune cell infiltration abundance and survival data of each patient for subsequent analysis.

### Differentially Expressed RNA Analysis and ceRNA Network Construction

The“limma” package of R software was utilized to identify the differentially expressed RNAs between cancer and normal tissues. Differentially expressed RNAs with | fold change (FC)| > 2 and *p* < 0.05 were considered statistically significant. Differentially expressed genes were visualized using the “Pheatmap” package of R software. Then, a ceRNA network between lncRNA-miRNA interactions and miRNA-mRNA interactions was constructed by using GDC RNA Tools. Cytoscape software was used to visualize the ceRNA regulatory network.

### Assessing the Prognosis-Related Genes in the ceRNA Network and Constructing a Prognostic Model

First, we merged the patient’s expression data and survival data. Univariate Cox regression analysis was used to identify prognosis-related genes in the ceRNA network of all CRC patients. Then, we utilized least absolute shrinkage and selection operator analysis(LASSO) Cox regression analysis to identify the genes with the best prognostic value. The expression of genes and the regression coefficients obtained in the regression model were used to calculate the patients’risk scores. The calculation formula is as follows.

$$\mathrm{Risk}\,\mathrm{score}\,\left(\mathrm{patients}\right)=\sum_{i=1}^{n}\mathrm{Coefficient}\,\left(\mathrm{gene}\,i\right)\times \mathrm{Expression}\mathrm{value}\,\left(\mathrm{gene}\,i\right)$$

Where *n*, *i*, coefficient, and expression valuerepresent the number of selected genes, gene number, regression coefficient value, and gene expression value, respectively. Based on the risk score, the patients were divided into high-and low-risk groups. The log-rank test was used to analyze the survival status of the two groups. The accuracy of the prognostic model was evaluated by receiver operating characteristic (ROC) curve analysis. In addition, we randomly divided all patients into two sets: validation set 1 and validation set 2 at a rate of 3:7. Using a previously obtained model, the randomly selected validation sets 1 and 2 were verified by the survival curve and ROC curve.

### Independent Prognostic Value of the Risk Model

Univariate and multivariate Cox regression analyses were performed to explore the independent prognostic value of the risk model. The risk score and different clinicopathologic features of each patient were used to predict the prognosis of patients with CRC. The “rms” R package was used to construct a nomogram to predict patient survival by using the independent prognostic factor. The calibration curve in the “survival” R package was used to assess the accuracy of the nomogram.

### Evaluation of Immune Cell Infiltration

TheCIBERSORT algorithm was used to calculate the tumor-infiltrating immune cells. The root mean squared error and *p*-value were counted for each sample file to improve the accuracy of the deconvolution algorithm. Only *p* < 0.05 was filtered and selected for further analysis, and the algorithm used a default signature matrix for 1,000-loop computation. The Wilcoxon rank-sum test was used to analyze the difference in the amount of immune cell infiltration in normal and tumor samples. The “Pheatmap” package was used to visualize the infiltration of immune cells in each sample. The Pearson correlation test was used to test the correlation of each type of immune cell. The correlation matrix was constructed based on the Pearson correlation coefficient to test the correlation of each type of immune cell by using R software.

### Correlation Analysis of Immune Cell Infiltration With Clinical Characteristics

Samples with accurate estimates of immune cell infiltration abundance were used for subsequent analysis. The clinical information of CRC patients in TCGA database, including survival time and survival status, was combined with the abundance of immune cell infiltration. Then, a survival analysis was performed on the filtered immune cells and the correlation between immune cell infiltration and clinicopathological characteristics was evaluated.

### Construction of a Prognostic Model by Tumor Immune Cell Infiltration

Constructing a prognostic model by tumor immune cell infiltration was similar to constructing a risk model using a ceRNA regulatory network. First, Kaplan-Meier survival analysis was used to evaluate the relationship between the abundance of immune cell infiltration and the overall survival of the patient. Then, univariate Cox regression analysis was used to identify prognosis-related infiltrating immune cell types. LASSO regression analysis was then performed to confirm the best candidate immune cell types to construct the model. Based on the Cox analysis, a nomogram was constructed with the regression coefficients.

### Statistical Analysis

Data were processed and analyzed by using Perl (5.30.1) and R (version 3.6.2) software. Kaplan-Meier survival curves with log-rank tests were applied for survival analysis. The Wilcoxon signed-rank test was used to identify differentially expressed RNAs.

## Results

### Identification of Differentially ExpressedGenes in CRC Patients and Establishmentof the ceRNA Network

**FIGURE 1:**
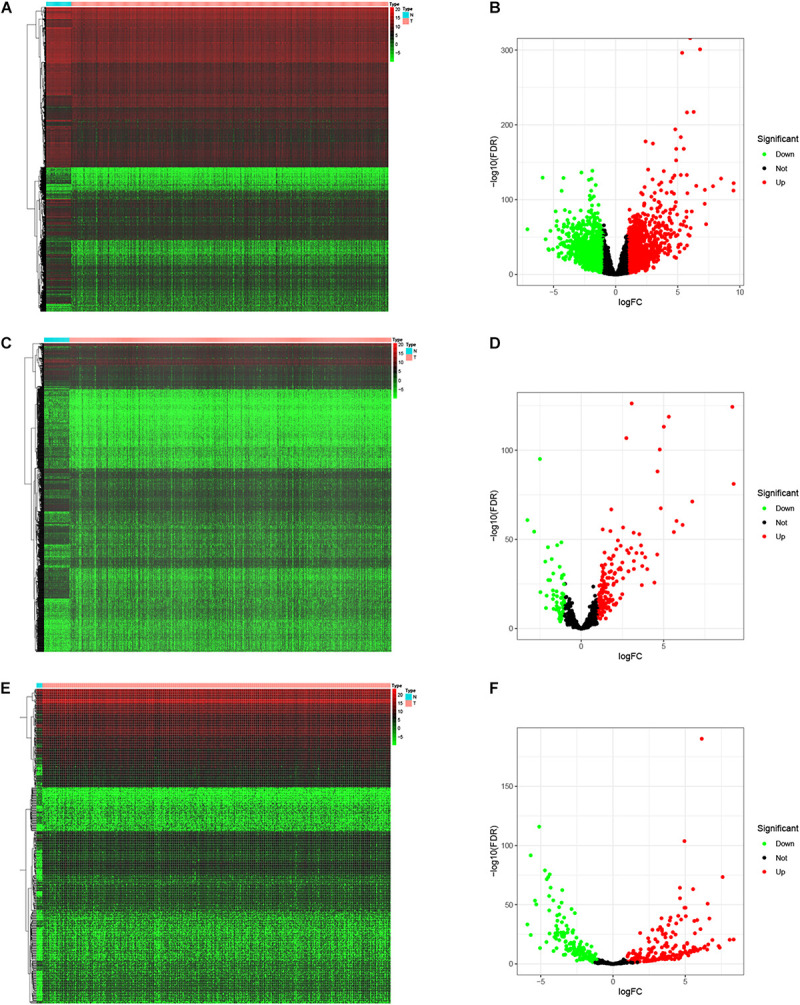
Differential expressed genes. The “limma” package of R was utilized to identify the differentially expressed genes. Heat map and Volcano map was plotted to visualize the differentially expressed mRNAs **(A,B)**, lncRNAs **(C,D)**, and miRNAs **(E,F)**. Red dots represent up-regulation and green dots represent down-regulation of differential expressed genes.

**FIGURE 2:**
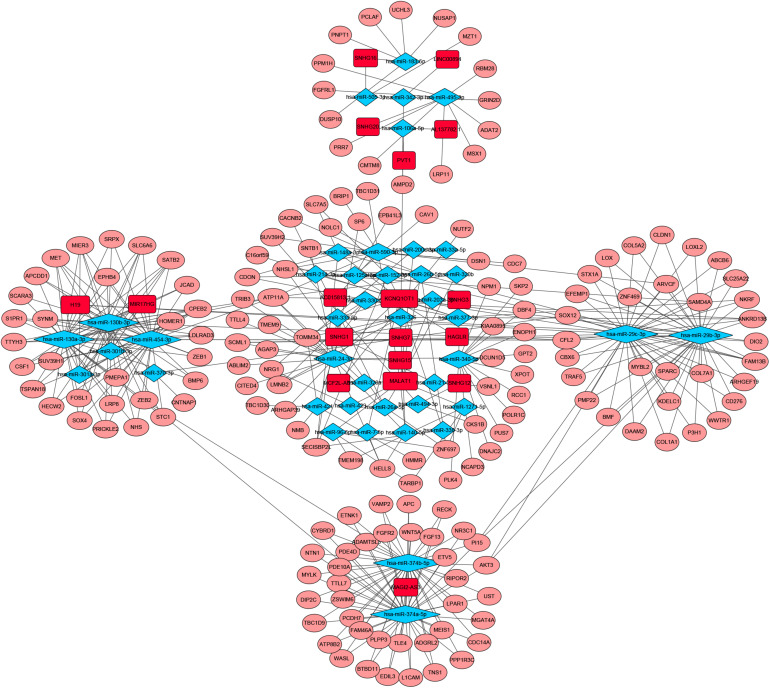
Competitive endogenous RNA (CeRNA) network of Differential expressed RNAs. GDC RNA Tools was used to construct ceRNA network. CeRNA regulatory network was visualized by using Cytoscape software. The red color genes represent differential expressed long noncoding RNAs(lncRNAs). The blue represent differential expressed miRNAs. The pink represent differential expressed mRNAs. Strings indicated the interaction among these genes. hazard ratio (HR) value >1 means that the gene is a high-risk gene, and HR < 1 means a low-risk gene.

To identify the differentially expressed genes involved in CRC, after the data were standardized through the “limma” package, we performed a differential analysis of transcriptome data. A total of 2,150 mRNAs and 1,028 lncRNAs were differentially expressed in 51 and 647 tumor samples. Among them, 945 mRNAs were downregulated and 1,205 were upregulated (Figures 1A,B) and 760 lncRNAs were upregulated and 268 were downregulated (Figures 1C,D). In addition, a total of 296 miRNAs were differentially expressed in 11 normal samples and 616 cancer samples, of which 201 were upregulated and 95 were downregulated, as shown in Figures 1E,F. Based on the database data contained in the R package and the prediction and calculation of the relationship between miRNA–mRNA and lncRNA–miRNA, we obtained the lncRNA–miRNA–mRNA regulatory network. Then, we combined the two relational pairs obtained above and visualized them by using Cytoscape software. There were 518 edges and 344 nodes in this network, including 244 mRNAs, 22 lncRNAs, and 78 miRNAs (Figure 2).

### Identify Prognostic-Related Genes in the ceRNA Network and Establish a Prognostic Model

**FIGURE 3:**
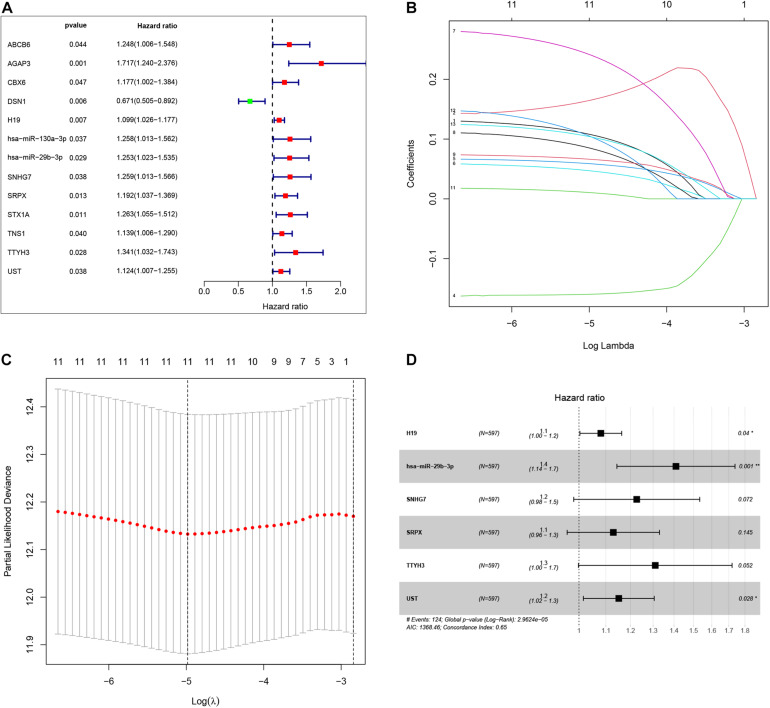
By using univariate Cox regression analysis, we identified thirteen survival-associated genes in the our ceRNA network **(A)**. Lasso coefficient profiles of the 13 prognosis-associated genes from the discovery dataset **(B)**. Partial likelihood deviance of variables revealed by the Lasso regression model **(C)**. Finally, six genes were included for the establishment of prognostic model **(D)**.

To identify the genes related to patient survival in the ceRNA network, we combined the survival status and survival time of each patient with gene expression data. A univariate Cox regression analysis identified thirteen candidate survival-associated genes (ABCB6, AGAP3, CBX6, DSN1, H19, hsa-miR-130a-3p, hsa-miR-29b-3p, SNHG7, SRPX, STX1A, TNS1, TTYH3, and UST) (Figure 3A). Then, we conducted LASSO regression analysis to determine the genetic model with the best prognostic value. The Lasso coefficient profile plot was produced against the log(*k*) sequence, and the minimized*k* method resulted in 11 optimal coefficients (Figures 3B,C). After filtering via the multivariate Cox regression analysis, we obtained a prognostic model based on six genes (H19, hsa-miR-29b-3p, SNHG7, SRPX, TTYH3, and UST) (Figure 3D). The correlation coefficients and hazard ratios of the genes in the model are shown in Table 2.

**TABLE 2:** Coefficients and multivariable Cox model results for competitive endogenous RNA(ceRNA) regulator network in CRC.

Gene symbol	Coef	HR	(95%CI)	*p*-value
H19	0.07739	1.080463	(1.003382–1.163467)	0.040426
hsa-miR-29b-3p	0.34341	1.143829	(1.143829–1.737485)	0.001282
SNHG7	0.204544	1.226966	(0.981693–1.53352)	0.072243
SRPX	0.121637	1.129344	(0.959015–1.329925)	0.144769
TTYH3	0.27013	1.310135	(0.99819–1.719566)	0.051546
UST	0.140996	1.15142	(1.015691–1.305286)	0.027576

**Note* coef, coefficient; HR, hazard ratio; CI, confidence interval.*

### Validation of the Predictive Performance of the Prognostic Model

**FIGURE 4:**
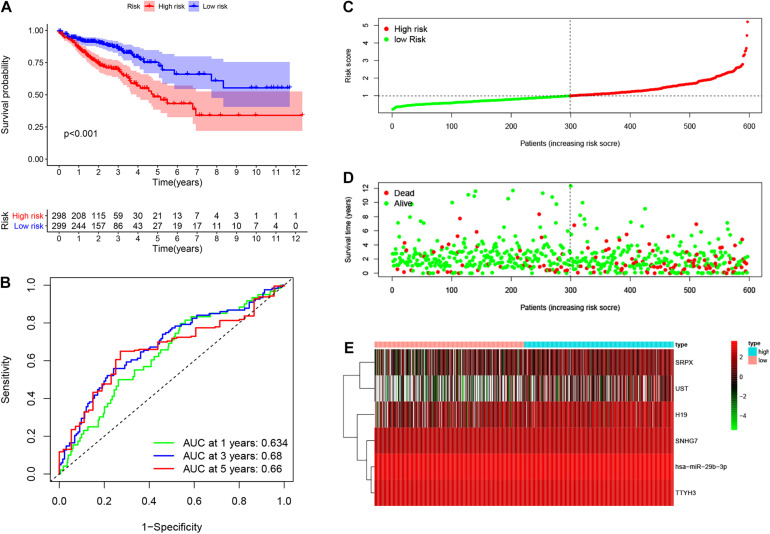
Performance of prognostic model. Log rank test was performed to plot survival probability of low-risk and high-risk patients **(A)**. Receiver operating characteristic (ROC) curve was constructed to verify the accuracy of the model **(B)**. The expression of genes and the regression coefficients obtained in the regression model were used to calculate the patients’ risk scores. Green dots represent risk score for low-risk patients; red dots represent risk score for high-risk patients **(C)**. The relationship between survival status and risk score. The abscissa represents the number of patients, and the ordinate is the risk score. Red dots represent dead patients, green dots are living patients **(D)**. Heatmap were plotted to identify the expression of six prognostic related genes between low risk and high risk colorectal cancer (CRC) patients **(E)**.

**FIGURE 5:**
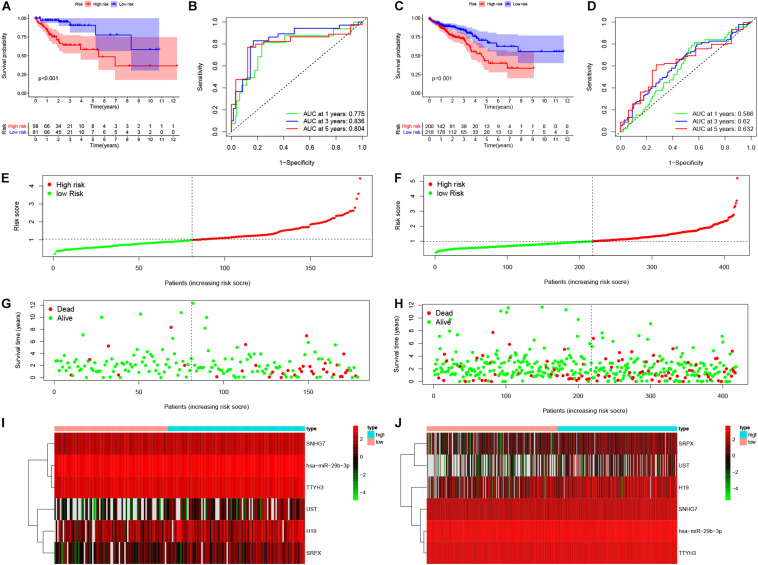
Validation of the prognostic model. Survival probability of low-risk and high-risk patients in validation set 1 and set 1, respectively **(A,C)**. ROC curve was constructed to verify prognostic value of the prognostic index in validation set 1 and set 1, respectively **(B,D)**. Risk ranking and the survival time based on risk ranking in two sets **(E–H)**. Heatmap was used to identify six prognostic related gene expression between low-risk and high-risk group in validation sets **(I,J)**.

Based on the median risk score in the risk model, patients were divided into a low-risk group (*n* = 299) and a high-risk group (*n* = 298). By using the log rank test, we plotted survival curves and found that patients in the low-risk group had better OS than those in the high-risk group (Figure 4A). The survival rates for the low-risk group and high-risk group were 86.2% (3-years),75.2% (5-years), 70.1% (3-years), and 48.5% (5-years), respectively. Then, a ROC curve was constructed to verify the accuracy of the model. The AUC value confirmed that the identified prognostic model was efficient for predicting OS in CRC patients (Figure 4B). According to the patient’s risk score, we ranked the patients and analyzed their distribution (Figure 4C). After sorting the patients by risk score, we observed that there were fewer deaths among patients in the low-risk group than the high-risk group. As the risk score increased, an increasing number of patients died. In addition, people in the low-risk group had longer survival times than those in the high-risk group (Figure 4D). The heat map shows that risk-related genes were overexpressed in the high-risk group of patients (Figure 4E). To further validate the predictive performance of the prognostic model, we randomly divided 597 patients into two subgroups: validation set 1 (dataset 1: *n* = 179) and validation set 2 (dataset 2: *n* = 418). We found that high-risk patients in the two subgroups had a better survival probability (*p* < 0.001) (Figures 5A,C). The AUC values in the two subgroups were 0.804 and 0.632 at 5 years (Figures 5B,D). After sorting the patients by risk score, we also observed that fewer patients died in the low-risk group than the high-risk group (Figures 5E–H). Similarly, the expression of six prognostic genes was higher among patients in the high-risk group (Figures 5I,J). Colon cancer and rectal cancer are reported to have different gene expression patterns (Gao et al., 2017). To further explore whether our prognostic model have a good performance in both of the colon cancer and rectal cancer, we grouped our samples into the colon cancer sub-group and rectal cancer sub-group. We found that our prognostic model has a good efficiency in both of these two groups. Our resultsfurtherillustratedthe prognostic model canbeappliedintodifferent types of colorectal cancer. The result was exhibited in Supplementary Figure 1. These results indicated that the predictive performance of the prognostic model is reliable in patients with CRC.

### Independent Prognostic Value of the Risk Model

**FIGURE 6:**
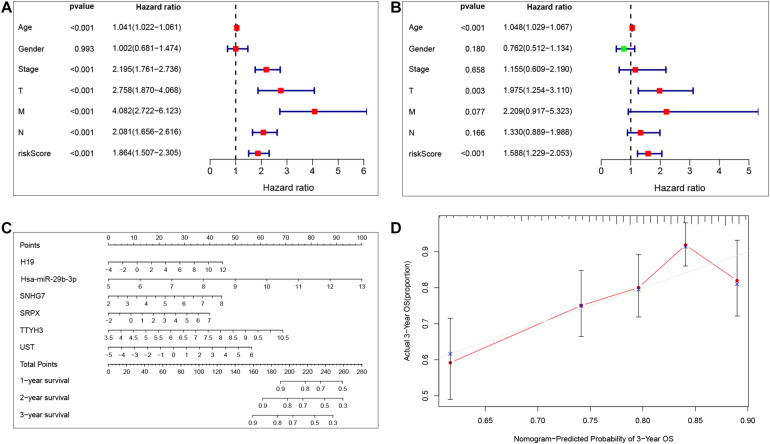
Independent prognostic value of the risk model. Univariate Cox regression analysis and multivariate Cox analysis was performed to verify the independent prognostic value of the risk model **(A,B)**. Nomogram graph was plotted to predict patients’ survival time **(C)**. Calibration curve of the nomogram (the horizontal and vertical axes represent the predicted survival probability and the actual survival probability, respectively; red segments separately represent the 3-years survival rate in the group with the highest consistency) **(D)**.

Our results show that the risk model can predict patient survival well. To further explore the independent prognostic value of the risk model, we first used a univariate Cox regression analysis to determine the hazard ratio of clinical features. We observed that T stage (*p* < 0.001), M stage (*p* < 0.001), *N*stage (*p* = 0.005), stage (*p* < 0.001), and risk score (*p* < 0.001) were significantly correlated with prognosis (Figure 6A). Then, we analyzed the independent prognostic value of the risk model by using multivariate analysis, and the results showed that stage (*p* < 0.001) and risk score (*p* < 0.001) were significantly correlated with prognosis (Figure 6B). These findings indicate that the risk score can be an independent prognostic biomarker for predicting patient survival. Meanwhile, to better predict the prognosis of patients with CRC at different years after diagnosis, we constructed a new nomogram based on gene expression. The higher the total score of the patient, the worse the prognosis (Figure 6C). The calibration curve confirms the accuracy of the nomogram (Figure 6D).

### Tumor Infiltrating Immune Cells in the TME

**FIGURE 7:**
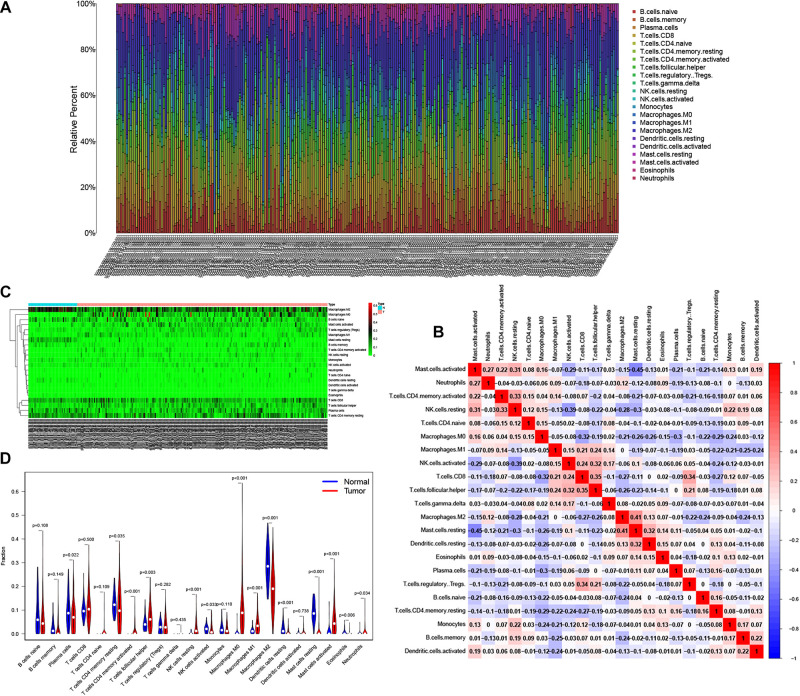
Tumor infiltrating immune cells in the tumor microenvironment (TME). CIBERSORT algorithm was used to calculate level of 22 Tumor infiltrating immune cells in CRC patients **(A)**. The correlation matrix of 22 types of Tumor infiltrating immune cells in CRC **(B)**. The “Pheatmap” package was used to visualize the infiltration of immune cells in CRC patients **(C)**. Difference in the proportion of 22 Tumor infiltrating immune cells between Normal tissue and Tumor tissue **(D)**.

**FIGURE 8:**
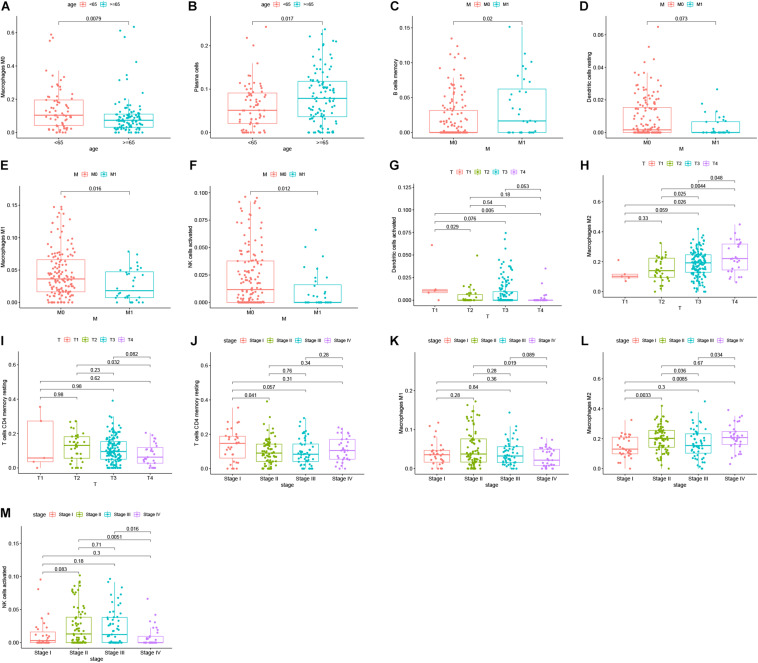
Survival analysis was performed to evaluate the correlation between immune cell infiltration and 152 clinicopathological characteristics. Correlation between immune cell infiltration and clinicopathologic features of patients **(A–M)**. The three horizontal lines in each picture means mean ± SD.

Reports have indicated that infiltrating immune cells in the TME are accompanied by cancer development (Zalocusky et al., 2018). Infiltrating immune cells are used as biomarkers for the immunotherapy response in many cancers (Chen et al., 2020). However, the function of every type of infiltrating immune cell in cancer development and the underlying mechanism still need further exploration. After removing samples that might be computationally inaccurate, we identified the proportion of 22 types of infiltrating immune cells in the 264 CRC patients mentioned above (Figure 7A). Then, we analyzed the correlation of all 22 immune infiltrating cells (Figure 7B). The heat map shows the amount of immune cell infiltration in the CRC samples (Figure 7C). Compared with normal tissue, the number of infiltrating immune cells, such as M2 macrophages, M0 macrophages, and B cells, was much higher. However, fewer immune infiltrating cells, such as resting mast cells, were observed in the tumor tissues (Figure 7D). In addition, we evaluated the relationship between immune infiltrating cells and patients’ clinical characteristics. We compared the number of infiltrating immune cells among patients with different clinical characteristics. We found that patients over 65 years old have more M0 macrophages and fewer plasma cells. Patients with different disease stages also had corresponding proportions of infiltrating immune cells (Figures 8A–M). These results indicated that infiltrating immune cells in the TME participated in the initiation and progression of cancer.

### Identification of Prognosis-Related Infiltrating Immune Cells and Establishment of a Prognostic Model

**FIGURE 9:**
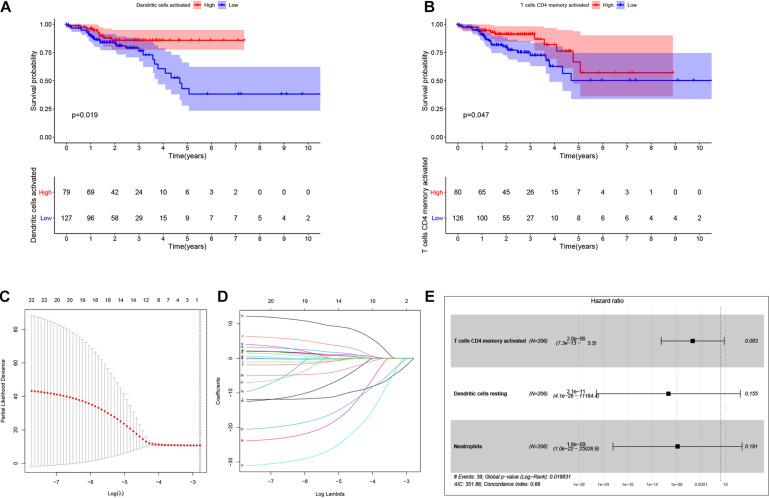
Identification of the prognostic related infiltrating immune cells and establish a prognostic model. Kaplan-Meier analysis was performed to identify survival probability of patients with different activation of Dendritic and T cells CD4 memory **(A,B)**. Lasso regression was conducted to identify prognostic related Tumor infiltrating immune cells. Lasso coefficient profiles of the 22 Tumor infiltrating immune cells. Partial likelihood deviance of variables revealed by the Lasso regression model **(C,D)**. Multivariate Logistic regression analysis of infiltrating immune cells. *p* < 0.05 indicates a significant correlation between genes and prognosis, HR value >1 means that the immune cells infiltrating is a high-risk gene, and HR < 1 means a low-risk immune cells infiltrating **(E)**.

**FIGURE 10:**
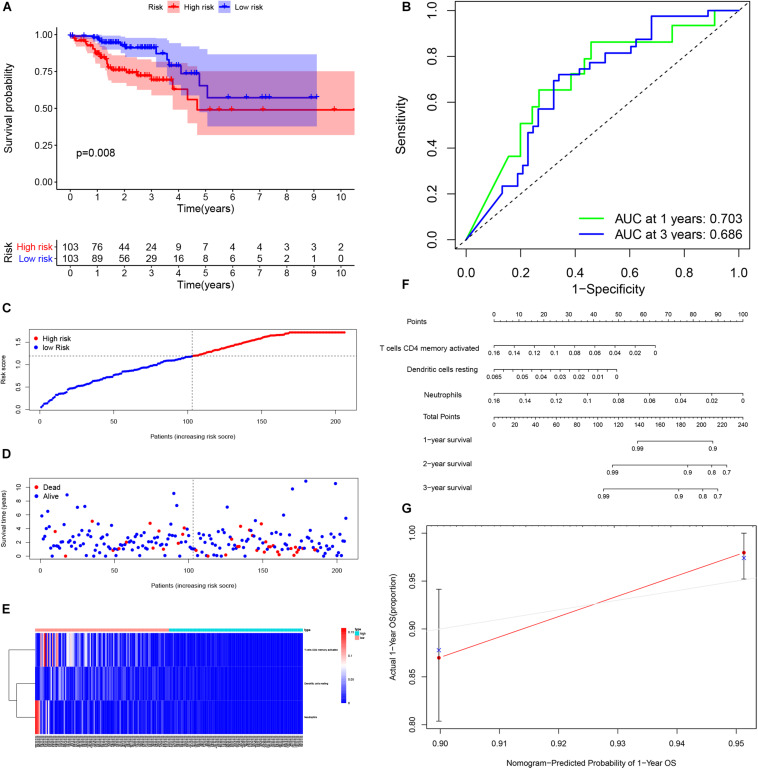
Performance of the infiltrating immune cells prognostic model. Survival analysis was plotted to visualize survival probability of low-risk and high-risk patients **(A)**. ROC curve was used to verify the accuracy of the model **(B)**. Distribution of groups based on the risk score **(C)**. Survival status of patients in different groups **(D)**. Heatmap was plotted to visualize 3 prognostic related infiltrating immune cells between low-risk and high-risk patients **(E)**. Nomogram graph was used to predict patients’ survival time **(F)**. Calibration curve of the nomogram **(G)**.

By using Kaplan-Meier analysis, we found that patients with lower proportions of dendritic cells and memory CD4 T cells were identified to have poor survival (Figures 9A,B). To identify prognosis-related infiltrating immune cells, we conducted LASSO regression and univariate Cox regression analyses by using the“glmnet” package in R software. The results showed that activated memory CD4 T cells, resting dendritic cells and neutrophils were superior in predicting patient prognosis (Figures 9C–E). Then, we constructed a risk model using the regression coefficient and infiltration of immune cells. Based on the results, patients were divided into a low-risk group (*n* = 103) and a high-risk group (*n* = 103). As the survival curve showed, patients in the high-risk group had significantly worse survival than patients in the low-risk group (Figure 10A). ROC curves were used to verify the accuracy of the model (Figure 10B). Then, according to the risk score, patients were ranked from low risk to high risk (Figure 10C). After ranking the patients by risk score, we observed that there were fewer deaths among patients in the low-risk group than among those in the high-risk group. As the risk score increased, more patients died (Figure 10D). In addition, patients with more tumor infiltrating immune cells had lower risk scores (Figure 10E). To more precisely predict the prognosis of patients with CRC at 1, 2, and 3 years, we constructed a new nomogram using OS-related variables (memory activated CD4 T cells, resting dendritic cells, and neutrophils). The nomogram results showed that the higher the level of immune cell infiltration, the better the survival rate of the patient (Figure 10F). The calibration curve confirms the accuracy of the nomogram (Figure 10G).

### Relationship Between the ceRNA Prognostic Model and the Infiltrating Immune Cell Prognostic Model

**FIGURE 11:**
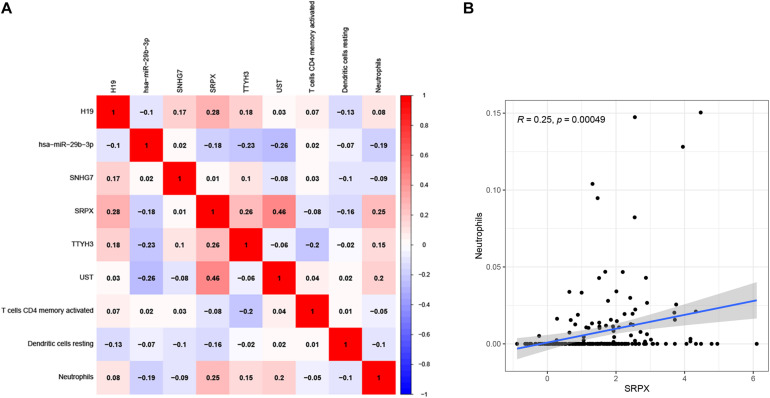
Correlation test was performed to explorecorrelation between ceRNA prognostic model and infiltrating immune cells prognostic model **(A)**. The correlation matrix of the elements in two prognostic models. The correlation between Neutrophils and SRPX **(B)**.

To determine the relationship between the two prognostic models, a correlation test was performed to explore the correlation between six prognostic genes and three prognostic infiltrating immune cells. We found that neutrophils were positively related to SRPX (Figures 11A,B), which might mean that the higher the expression of SRPX, the higher the infiltration abundance of immune cells. These two indicators may serve as prognostic biomarkers for CRC patients and could eventually be applied to formulate personalized targeted therapy.

## Discussion

Colorectal cancer is one of the most common malignant tumors in humans. Despite continuous advancements in diagnosis and treatment, CRC accounts for approximately 10% of all cancers and cancer-related deaths worldwide each year. Biomarkers have crucial functions in the diagnosis and treatment of cancer patients, including CRC patients. For cancer patients with early-stage disease, biomarkers can be used as a detection method to identify susceptibility or early-stage disease. For patients with advanced cancer, biomarkers can be used as therapeutic targets to improve the survival time of patients. Although relevant biomarkers have been identified in CRC, more effective biomarkers for the future development of prognosis in CRC are needed.

In this study, we constructed a ceRNA network prognostic model and an infiltrating immune cell prognostic model. First, the expression profiles of 597 patients based on TCGA data were obtained. The differential expression of 1,028 lncRNAs, 2,150 mRNAs, and 296 miRNAs were identified. A ceRNA network among these differentially expressed genes was constructed using GDC RNA Tools. Then, we integrated matched clinical EC data and obtained thirteen candidate survival-associated genes. The“Glmnet” package was used to identify the six genes with the best prognostic value (H19, hsa-miR-29b-3p, SNHG7, SRPX, TTYH3, and UST) and establish the prognostic model. Most of these genes have been reported to regulate cancer progression. Yang et al. (2018) reported that lncRNA H19 promotes the migration and invasion of colon cancer cells by activating the MAPK signaling pathway. In addition, Ding et al. (2018) found that the lncRNA H19/miR-29b-3p/PGRN axis promoted the EMT in CRC cells by acting on Wnt signaling. Shan et al. (2018) reported that lncRNA SNHG7 can function as a ceRNA to promote the proliferation and liver metastasis of CRC. Yang Li also found that lncRNA SNHG7 played an oncogenic role by regulating the PI3K/Akt/mTOR pathway by competing for endogenous miR-34a and GALNT7 in CRC (Li et al., 2018). SNHG7 has also been identified as a gene with potential prognostic and diagnostic value in CRC (Hu et al., 2019). These findings suggest that genes in our prognostic model play crucial roles in CRC. In addition, the prognostic model was proved to have a good efficiency in colon cancer and rectal cancer, respectively. In our prognostic model, the 1-, 2-, and 3-year survival rates for CRC patients can be predicted based on the risk score. The AUC value confirmed the efficiency of our prognostic model in predicting OS for CRC patients. The accuracy of the model was also tested in two subgroups. Then, we used univariate and multivariate regression analyses and found that the prognostic model can be an independent prognostic biomarker in predicting patient survival outcomes. These results indicated that our prognostic model functions in CRC patients.

The immune system plays an important role in the development of cancer. Mounting evidence shows that the TME, including macrophages, neutrophils and T cells, plays a crucial role in the occurrence and development of cancer, including CRC. The TME, especially cancer-infiltrating immune cells, has been reported to participate in cancer prognosis (Li et al., 2017). The composition of tumor infiltrating immune cells (TIICs)varies with the immune status of the host and has potential prognostic value (Sato et al., 2013; Zhang et al., 2019). A recent report indicated that ceRNA network is associated with immune infiltration in colorectal cancer (Chen et al., 2021). They identified prognosis related lncRNAs and analyzed the relationship between the expression of mir4435-2hg, ELFN1-AS1 and immune infiltration. Our result also revealed the correlation between ceRNA network and immune infiltration, but in our prognostic model, we identified all the prognostic related lncRNAs, mRNAs, and miRNAs in the ceRNA network for the first time. We established the prognostic model by lasso regression. The prognostic model was further confirmed be used as an independent prognostic indicator. In addition, we identified prognostic related immune infiltration cells and constructed the immune infiltration model. The results showed that the proportion of tumor-infiltrating immune cells (activated memory CD4 T cells, resting dendritic cells, and neutrophils) was negatively correlated with the patient risk score. ROC curves were tested to verify the accuracy of the model. In addition, we determined the relationship between the ceRNA prognostic model and the infiltrating immune cell prognostic model. Neutrophilswere positively related to SRPX, which may indicate that the higher the expression of this gene, the higher the degree of immune cell infiltration. SRPX has been reported to regulate cancer-associated fibroblasts to promote the invasiveness of ovarian carcinoma (Liu et al., 2019). However, whether SRPX can modulate immunity to control cancer progression and whether it can replace the depth of neutrophil infiltration remain unclear, and further research is needed.

In summary, our study constructed a prognostic model of the ceRNA network and an infiltrating immune cell prognostic model for CRC. These two models can accurately predict the prognosis of patients with CRC. These results provide insights for predicting the prognosis of cancer patients and might have crucial value for exploring new cancer diagnosis methods and treatment strategies.

## Data Availability Statement

The original contributions presented in the study are included in the article/Supplementary Material, further inquiries can be directed to the corresponding author.

## Author Contributions

XG, YW, and XL designed the study. XG, XL, CQ, and HZ collected and analyzed the data. XL, XG, and AC wrote and revised the manuscript. ZW was responsible for supervising the study. All authors read and gave final approval of the manuscript.

## Conflict of Interest

The authors declare that the research was conducted in the absence of any commercial or financial relationships that could be construed as a potential conflict of interest.

## Footnotes

1 https://cibersort.stanford.edu/

## References

[B1] Belli C. Trapani D. Viale G. D’Amico P. Duso B. A. Della Vigna P. (2018). Targeting the microenvironment in solid tumors. *Cancer Treat. Rev.* 65 22–32. 10.1016/j.ctrv.2018.02.004 29502037

[B2] Chai J. Zhang J. Han D. Dong W. Han L. Zou L. (2020). Identification of long non-coding RNA SCARNA9L as a novel molecular target for colorectal cancer. *Oncol. Lett.* 20 1452–1461. 10.3892/ol.2020.11661 32724388 PMC7377086

[B3] Chao Y. Zhou D. (2019). lncRNA-D16366 is a potential biomarker for diagnosis and prognosis of hepatocellular carcinoma. *Med. Sci. Monit.* 25 6581–6586. 10.12659/msm.915100 31475695 PMC6738002

[B4] Chen J. Song Y. Li M. Zhang Y. Lin T. Sun J. (2021). Comprehensive analysis of ceRNA networks reveals prognostic lncRNAs related to immune infiltration in colorectal cancer. *BMC Cancer* 21:255. 10.1186/s12885-021-07995-2 33750326 PMC7941714

[B5] Chen Y. Zhao B. Wang X. (2020). Tumor infiltrating immune cells (TIICs) as a biomarker for prognosis benefits in patients with osteosarcoma. *BMC Cancer* 20:1022. 10.1186/s12885-020-07536-3 33087099 PMC7579940

[B6] De Robertis M. Mazza T. Fusilli C. Loiacono L. Poeta M. L. Sanchez M. (2018). EphB2 stem-related and EphA2 progression-related miRNA-based networks in progressive stages of CRC evolution: clinical significance and potential miRNA drivers. *Mol. Cancer* 17:169. 10.1186/s12943-018-0912-z 30501625 PMC6271583

[B7] Ding D. Li C. Zhao T. Li D. Yang L. Zhang B. (2018). LncRNA H19/miR-29b-3p/PGRN axis promoted epithelial-mesenchymal transition of colorectal cancer cells by acting on wnt signaling. *Mol. Cells* 41 423–435. 10.14348/molcells.2018.2258 29754471 PMC5974619

[B8] Gao X. H. Yu G. Y. Gong H. F. Liu L. J. Xu Y. Hao L. Q. (2017). Differences of protein expression profiles, KRAS and BRAF mutation, and prognosis in right-sided colon, left-sided colon and rectal cancer. *Sci. Rep.* 7:7882. 10.1038/s41598-017-08413-z 28801584 PMC5554205

[B9] Hu Y. Wang L. Li Z. Wan Z. Shao M. Wu S. (2019). Potential prognostic and diagnostic values of CDC6, CDC45, ORC6 and SNHG7 in colorectal cancer. *Onco Targets Ther.* 12 11609–11621. 10.2147/ott.S231941 32021241 PMC6942537

[B10] Jochems C. Schlom J. (2011). Tumor-infiltrating immune cells and prognosis: the potential link between conventional cancer therapy and immunity. *Exp. Biol. Med.* 236 567–579. 10.1258/ebm.2011.011007 21486861 PMC3229261

[B11] Koulis C. Yap R. Engel R. Jardé T. Wilkins S. Solon G. (2020). Personalized medicine-current and emerging predictive and prognostic biomarkers in colorectal cancer. *Cancers* 12:812. 10.3390/cancers12040812 32231042 PMC7225926

[B12] Krempski J. Karyampudi L. Behrens M. D. Erskine C. L. Hartmann L. Dong H. (2011). Tumor-infiltrating programmed death receptor-1+ dendritic cells mediate immune suppression in ovarian cancer. *J. Immunol.* 186 6905–6913. 10.4049/jimmunol.1100274 21551365 PMC3110549

[B13] Ladányi A. (2015). Prognostic and predictive significance of immune cells infiltrating cutaneous melanoma. *Pigment Cell Melanoma Res.* 28 490–500. 10.1111/pcmr.12371 25818762

[B14] Le K. Guo H. Zhang Q. Huang X. Xu M. Huang Z. (2019). Gene and lncRNA co-expression network analysis reveals novel ceRNA network for triple-negative breast cancer. *Sci. Rep.* 9:15122. 10.1038/s41598-019-51626-7 31641220 PMC6805880

[B15] Lee S. S. Cheah Y. K. (2019). The interplay between MicroRNAs and cellular components of tumour microenvironment (TME) on non-small-cell lung cancer (NSCLC) progression. *J. Immunol. Res.* 2019:3046379. 10.1155/2019/3046379 30944831 PMC6421779

[B16] Li C. Liu T. Zhang Y. Li Q. Jin L. K. (2019). LncRNA-ZDHHC8P1 promotes the progression and metastasis of colorectal cancer by targeting miR-34a. *Eur. Rev. Med. Pharmacol. Sci.* 23 1476–1486. 10.26355/eurrev_201902_17105 30840269

[B17] Li T. Fan J. Wang B. Traugh N. Chen Q. Liu J. S. (2017). TIMER: a web server for comprehensive analysis of tumor-infiltrating immune cells. *Cancer Res.* 77 e108–e110. 10.1158/0008-5472.Can-17-0307 29092952 PMC6042652

[B18] Li Y. Zeng C. Hu J. Pan Y. Shan Y. Liu B. (2018). Long non-coding RNA-SNHG7 acts as a target of miR-34a to increase GALNT7 level and regulate PI3K/Akt/mTOR pathway in colorectal cancer progression. *J. Hematol. Oncol.* 11:89. 10.1186/s13045-018-0632-2 29970122 PMC6029165

[B19] Liu C. L. Pan H. W. Torng P. L. Fan M. H. Mao T. L. (2019). SRPX and HMCN1 regulate cancer-associated fibroblasts to promote the invasiveness of ovarian carcinoma. *Oncol. Rep.* 42 2706–2715. 10.3892/or.2019.7379 31638245

[B20] Liu X. Wu S. Yang Y. Zhao M. Zhu G. Hou Z. (2017). The prognostic landscape of tumor-infiltrating immune cell and immunomodulators in lung cancer. *Biomed. Pharmacother.* 95 55–61. 10.1016/j.biopha.2017.08.003 28826097

[B21] Long J. Bai Y. Yang X. Lin J. Yang X. Wang D. (2019). Construction and comprehensive analysis of a ceRNA network to reveal potential prognostic biomarkers for hepatocellular carcinoma. *Cancer Cell Int.* 19:90. 10.1186/s12935-019-0817-y 31007608 PMC6458652

[B22] Ni W. Yao S. Zhou Y. Liu Y. Huang P. Zhou A. (2019). Long noncoding RNA GAS5 inhibits progression of colorectal cancer by interacting with and triggering YAP phosphorylation and degradation and is negatively regulated by the m(6)A reader YTHDF3. *Mol. Cancer* 18:143. 10.1186/s12943-019-1079-y 31619268 PMC6794841

[B23] Patnaik S. Anupriya P. (2019). Drugs targeting epigenetic modifications and plausible therapeutic strategies against colorectal cancer. *Front. Pharmacol.* 10:588. 10.3389/fphar.2019.00588 31244652 PMC6563763

[B24] Picardo F. RoRomanelli A. Muinelo-Romay L. Mazza T. Fusilli C. Parrella P. (2019). Diagnostic and prognostic value of B4GALT1 hypermethylation and its clinical significance as a novel circulating cell-free dna biomarker in colorectal cancer. *Cancers* 11:1598. 10.3390/cancers11101598 31635093 PMC6826707

[B25] Rodriguez-Casanova A. Costa-Fraga N. Bao-Caamano A. López-López R. Muinelo-Romay L. Diaz-Lagares A. (2021). Epigenetic landscape of liquid biopsy in colorectal cancer. *Front. Cell Dev. Biol.* 9:622459. 10.3389/fcell.2021.622459 33614651 PMC7892964

[B26] Sato Y. Yoshizato T. Shiraishi Y. Maekawa S. Okuno Y. Kamura T. (2013). Integrated molecular analysis of clear-cell renal cell carcinoma. *Nat. Genet.* 45 860–867. 10.1038/ng.2699 23797736

[B27] Shan Y. Ma J. Pan Y. Hu J. Liu B. Jia L. (2018). LncRNA SNHG7 sponges miR-216b to promote proliferation and liver metastasis of colorectal cancer through upregulating GALNT1. *Cell Death Dis.* 9:722. 10.1038/s41419-018-0759-7 29915311 PMC6006356

[B28] Sung H. Ferlay J. Siegel R. L. Laversanne M. Soerjomataram I. Jemal A. (2021). Global cancer statistics 2020: globocan estimates of incidence and mortality worldwide for 36 cancers in 185 countries. *CA Cancer J. Clin.* 71 209–249. 10.3322/caac.21660 33538338

[B29] Terrén I. Orrantia A. Vitallé J. Zenarruzabeitia O. Borrego F. (2019). NK cell metabolism and tumor microenvironment. *Front. Immunol.* 10:2278. 10.3389/fimmu.2019.02278 31616440 PMC6769035

[B30] Wang L. Cho K. B. Li Y. Tao G. Xie Z. Guo B. (2019). Long noncoding RNA (lncRNA)-mediated competing endogenous rna networks provide novel potential biomarkers and therapeutic targets for colorectal cancer. *Int. J. Mol. Sci.* 20:5758. 10.3390/ijms20225758 31744051 PMC6888455

[B31] Wei L. Wang X. Lv L. Zheng Y. Zhang N. Yang M. (2019). The emerging role of noncoding RNAs in colorectal cancer chemoresistance. *Cell Oncol.* 42 757–768. 10.1007/s13402-019-00466-8 31359293 PMC12994294

[B32] Xiao H. Zhang F. Zou Y. Li J. Liu Y. Huang W. (2018). The function and mechanism of long non-coding RNA-ATB in cancers. *Front. Physiol.* 9:321. 10.3389/fphys.2018.00321 29692736 PMC5902489

[B33] Xu W. Zhou G. Wang H. Liu Y. Chen B. Chen W. (2020). Circulating lncRNA SNHG11 as a novel biomarker for early diagnosis and prognosis of colorectal cancer. *Int. J. Cancer* 146 2901–2912. 10.1002/ijc.32747 31633800

[B34] Yang W. Redpath R. E. Zhang C. Ning N. (2018). Long non-coding RNA H19 promotes the migration and invasion of colon cancer cells via MAPK signaling pathway. *Oncol. Lett.* 16 3365–3372. 10.3892/ol.2018.9052 30127936 PMC6096146

[B35] Yu M. Song X. G. Zhao Y. J. Dong X. H. Niu L. M. Zhang Z. J. (2021). Circulating serum exosomal long non-coding RNAs FOXD2-AS1, NRIR, and XLOC_009459 as diagnostic biomarkers for colorectal cancer. *Front. Oncol.* 11:618967. 10.3389/fonc.2021.618967 33777763 PMC7996089

[B36] Zalocusky K. A. Kan M. J. Hu Z. Dunn P. Thomson E. Wiser J. (2018). The 10,000 immunomes project: building a resource for human immunology. *Cell Rep.* 25 513–522. 10.1016/j.celrep.2018.09.021 30304689 PMC6263160

[B37] Zhang S. Zhang E. Long J. Hu Z. Peng J. Liu L. (2019). Immune infiltration in renal cell carcinoma. *Cancer Sci.* 110 1564–1572. 10.1111/cas.13996 30861269 PMC6501001

