# Supplementary figures and images for: Construction and Comprehensive Prognostic Analysis of a lncRNA–miRNA–mRNA Regulatory Network and Tumor Immune Cell Infiltration in Colorectal Cancer
Source: Front Genet. 2021 Jul 1;12:652601. doi: 10.3389/fgene.2021.652601 (PMC8281064; doi:10.3389/fgene.2021.652601)

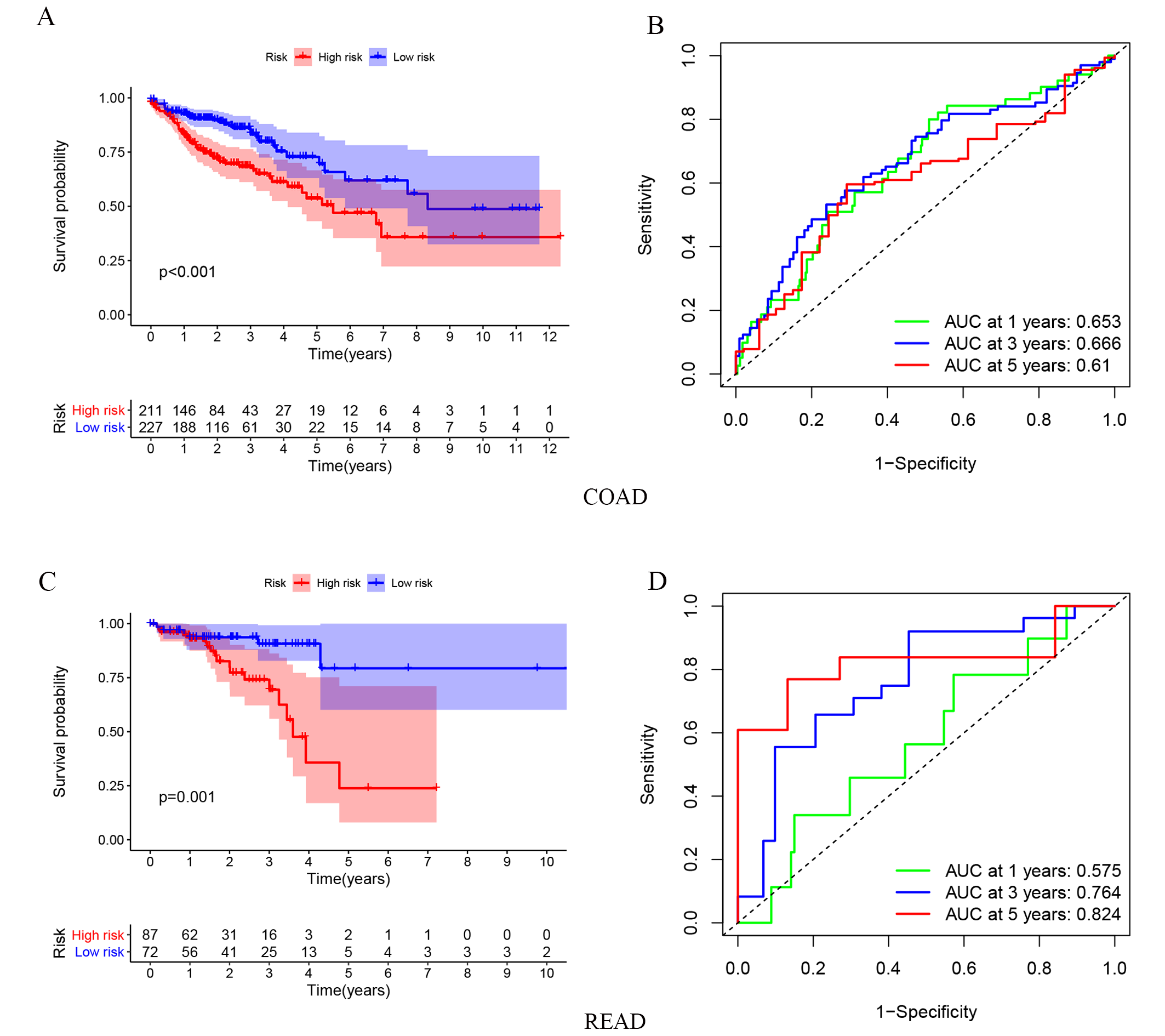

Supplement: Supplementary file 1 [file Image_1.TIF]
